# High-Risk Chronic Lymphocytic Leukemia in a Young Adult Treated With Reduced-Dosage, Fixed-Duration Ibrutinib and Venetoclax

**DOI:** 10.14740/jmc5237

**Published:** 2026-03-04

**Authors:** Caryn Louise D. Gutierrez, Anne Kristine H. Quero-Taggaoa, Timothy Carl F. Uy, Januario Antonio D. Veloso

**Affiliations:** a Department of Internal Medicine, Division of Hematology, Philippine General Hospital, Manila, Philippines; b Department of Pathology and Laboratory Medicine, Philippine General Hospital, Manila, Philippines

**Keywords:** Chronic lymphocytic leukemia, High-risk CLL, Young adult, Fixed-duration therapy, Reduced dosage, Targeted therapy, Resource-limited setting

## Abstract

Chronic lymphocytic leukemia (CLL) is a disease primarily of the elderly; however, in rare cases, it can occur among adolescents and young adults (AYAs). While evidence-based guidelines are established, the guidelines include elderly with very limited data among young adults. CLL in AYA is deemed as high-risk disease. They experience longer survival than their older age counterparts, but this longevity increases their susceptibility to secondary cancers, CLL-related complications, and treatment-related adverse events. Thus, effective treatment requires a careful balance of efficacy and safety. A 32-year-old female presented with multiple neck masses, night sweats, weight loss, and easy fatigability. Workup revealed bicytopenia (anemia and thrombocytopenia). A cervical lymph node biopsy was compatible with small lymphocytic lymphoma (SLL); however, bone marrow aspiration with biopsy was done and signed out as CLL. Due to overlapping immuno-morphologic features, and her age, B-cell acute lymphoblastic leukemia cannot be entirely ruled out. Further testing with flow cytometry for basic leukemia panel and histopathology with immunohistochemistry favored a diagnosis of CLL. She was started on a reduced-dosage, fixed-duration ibrutinib and venetoclax. Since treatment initiation, patient had significant symptom improvement and no longer require blood transfusion. The report emphasized the need for individualized therapy to enhance outcomes and quality of life in young CLL patients. It discussed a rare case of a young adult with CLL, highlighting its diagnostic challenges, treatment, and outcomes. A fixed-duration, lower-dose regimen of ibrutinib and venetoclax appears to be a promising, safe, and effective treatment for CLL in young adults.

## Introduction

Chronic lymphocytic leukemia (CLL) is an indolent malignancy characterized by the clonal proliferation of mature but immunologically dysfunctional B-cell lymphocytes in the peripheral blood [1]. The exact etiology remains unclear; however, genetic predisposition appears to play a central role, with additional risk factors including chemical exposure, radiation, and smoking [1]. CLL predominantly affects older adults, with a median age at diagnosis of approximately 70 years [2]. In contrast, its occurrence in adolescents and young adults (AYAs), typically defined as individuals aged 15–39 years, is exceedingly rare, accounting for less than 1% of cases [3, 4].

CLL is frequently diagnosed incidentally when patients undergo evaluation for unrelated conditions. Routine laboratory investigations, particularly complete blood count (CBC), may reveal leukocytosis with markedly elevated absolute lymphocyte count (ALC) [5]. Although many patients are asymptomatic at diagnosis, progressive disease may lead to constitutional symptoms such as fever, anorexia, weight loss, lymphadenopathy, hepatosplenomegaly, and exertional dyspnea [5].

Given its rarity in younger patients, the management of CLL in the AYA population poses distinct challenges, including a higher prevalence of unfavorable prognostic features and historically limited therapeutic options [6, 7]. In recent years, however, the treatment landscape of CLL has been transformed by the advent of targeted agents, which are increasingly favored over traditional chemoimmunotherapy across treatment settings. Because younger patients have a longer life expectancy and are at risk for cumulative disease- and treatment-related toxicities, fixed-duration regimens—such as venetoclax-based therapies—represent an important therapeutic consideration [8, 9]. Here, we report a case of CLL in a young adult treated with a reduced-dose, fixed-duration regimen of ibrutinib and venetoclax.

## Case Report

A 32-year-old Filipino female, a non-smoker with no known comorbidities, who worked as a factory worker in a plastic molding company, presented with multiple neck masses. She had no family history of CLL/small lymphocytic lymphoma (SLL) or Waldenstrom macroglobulinemia. She had a 6-month history of progressively enlarging left lateral neck mass associated with night sweats, undocumented weight loss, and easy fatigability.

### Investigations

She sought consultation with a local physician, where CBC was initially done revealing anemia with hemoglobin of 50 g/L, hematocrit of 19%, white blood cell (WBC) of 13,200 cells/mm^3^ with lymphocytic predominance (ALC 10,652 cells/mm^3^), and a platelet count of 95,000 cells/mm^3^. Blood transfusion of packed red blood cells was done, and neck mass biopsy was facilitated revealing atypical lymphoid proliferation. Immunohistochemistry (IHC) staining result showed strong positivity for CD20, CD5, BCL2, and CD23, with negative staining for CD3, CD10, cyclin D1, and low Ki67 of about 20-30% in the cells of interest. This was signed out as SLL. On subsequent follow-up, she was noted to have recurrent anemia requiring transfusion once or twice weekly. Workup for immune-mediated hemolytic anemia was done: the direct antiglobulin test was negative; total bilirubin was within normal range at 0.84 mg/dL, with direct bilirubin at 0.29 mg/dL and indirect bilirubin at 0.55 mg/dL. Lactate dehydrogenase and uric acid were slightly elevated at 310 U/L and 7.57 mg/dL, respectively.

**Figure 1:**
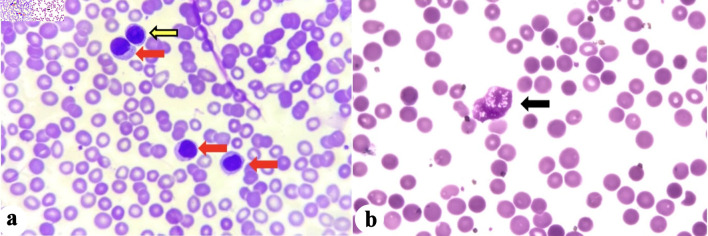
(a) Mature lymphocytes (red arrows), and prolymphocyte (yellow arrow). (b) Basket cell/smudge cell (black arrow).

Peripheral blood smear (PBS) was done and showed predominance of moderate-sized mononuclear cells with condensed chromatin, and moderate amount of basophilic cytoplasm compatible with mature lymphocytes (Fig. 1). There were some moderate-sized mononuclear cells with slightly clumped chromatin, single prominent nucleoli, and with scanty basophilic cytoplasm compatible with prolymphocytes. Some remnants of cells that lack any identifiable cytoplasmic membrane or nuclear structure (“smudge or basket cells”) were also identified.

**Figure 2:**
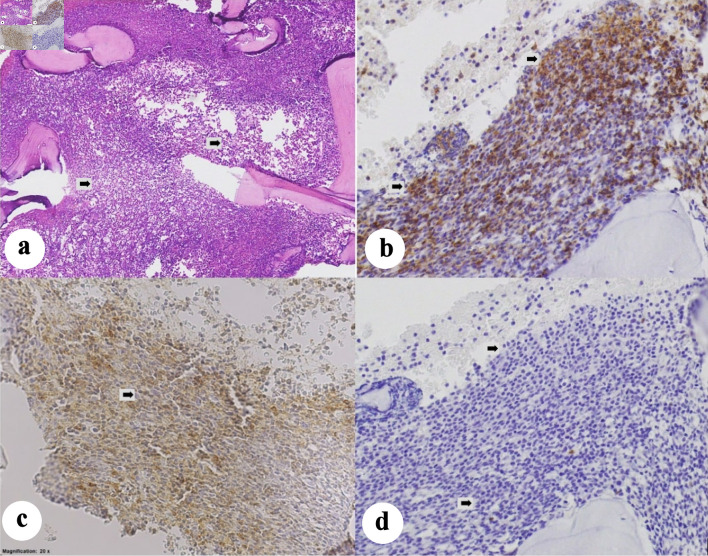
(a) Hypercellular bone marrow partially effaced by a population of atypical round cells that comprise approximately 80–90% of the marrow cellularity (arrows). The atypical cells show strong membranous staining for CD20 (b), moderate-to-strong membranous staining for CD5 (c) and are negative for cyclin-D1 (d). Taken together, the immuno-morphologic features support involvement by chronic lymphocytic leukemia (arrows).

A baseline positron emission tomography/computed tomography (PET/CT) scan was ordered for staging, which revealed multiple lymphadenopathies in the neck, chest, abdomen, pelvis, and lower extremity with the largest aggregate measuring 16 × 10.6 × 6.8 cm in the retroperitoneal region (Deaville 4) (reference maximum standardized uptake value (SUVmax): liver 2.7, mediastinum 1.7). The spleen was markedly enlarged measuring 13.2 × 10.8 × 7.5 cm (splenic index 1,069). The liver was likewise enlarged, measuring 16.3 cm, with homogenous parenchyma and smooth margins. There were also clusters of hypermetabolic pulmonary nodules and diffuse hypermetabolism in the marrow of almost the entire skeleton, which was interpreted as reactive vs. lymphomatous disease. Since the patient was a young adult presenting with recurrent anemia and thrombocytopenia, and PBS showed medium-sized cells as described with relatively moderate amount of cytoplasm compared with what is typical for SLL or CLL, a bone marrow aspiration (BMA) with biopsy was done. BMA specimens were sent for histopathology and flow cytometry for basic leukemia panel. Histopathology revealed a hypercellular marrow with 95–98% cellularity (Fig. 2). There was a population of medium-to-large cells with fine chromatin, prominent nucleoli, and high nuclear-to-cytoplasmic ratios, comprising 90–95% of nucleated cells. IHC staining result showed strong positivity in CD5, and CD20, with negative cyclin D1, CD3, CD34, and TdT in the cells of interest. This result was signed out as CLL. The flow cytometry results showed low-to-intermediate side scatter with bright CD45. Immaturity markers were negative for CD34 but showed bright human leukocyte antigen-DR isotype (HLA-DR). The cells of interest exhibited moderately bright positivity in B-cell markers (CD19, CD20, and cCd79a), and bright positivity in T-cell marker (CD5). However, there were no significant expressions of surface immunoglobulins (kappa and lambda). Overall, these findings were consistent with a mature B-cell neoplasm, specifically B-cell CLL. However, given the overlapping immuno-morphologic features, B-lymphoblastic leukemia cannot be totally excluded. Additional IHCs requested showed moderately bright CD200 and CD23, and no significant expression on FMC7. Hence, with this result, a diagnosis of CLL was finally made. Fluorescence *in situ* hybridization (FISH) panel for CLL was then requested, which revealed the presence of TP53 deletion, and ATM (11q22) deletion. Chromosomal abnormalities included deletion in 13q14, 17p13.1, and 11q22. With the above-mentioned findings, she was risk-stratified as CLL, Rai stage IV (high risk).

### Treatment

At first, the intention was to initiate treatment with a Bruton tyrosine kinase inhibitor (BTKi), which the patient was amenable to. However, she brought up her immediate concerns which were the duration of therapy, long-term side effects of the medication, and over-all cost of treatment as a maintenance therapy. Since the patient was young, financially constrained, and not amenable to have maintenance therapy for her condition, she was treated with a fixed-duration, reduced-dose regimen of ibrutinib and venetoclax. Her treatment regimen was a 28-day cycle of ibrutinib 420 mg daily for 3 months, followed by dose reduction to 280 mg daily for 1 month, and eventually maintained to 140 mg daily. Venetoclax was started initially in the fourth cycle of therapy at 100 mg on alternate days for 2 weeks and was subsequently increased only up to 100 mg daily due to recurrent anemia. She was also on febuxostat 40 mg tablet daily for the hyperuricemia. During the course of treatment, there were no treatment-related adverse events noted.

### Follow-up and outcomes

Interim PET/CT scan with contrast after three cycles of ibrutinib and venetoclax revealed a significant decrease in the cervical lymphadenopathies from 2.4 cm to largest lymph node measuring 0.8 cm, and her axillary lymphadenopathies likewise decreased in size from 3.1 cm to largest being 1 cm. The multiple enlarged lymph nodes in the abdomen initially with the largest aggregate measuring 16 × 10.6 × 6.8 cm now decreased significantly to 3 × 1.5 cm. Her liver was still enlarged but decreased from 16.3 cm to 15.3 cm, her splenic index also decreased from 1,069 to 717. There was a significant decrease in marrow heterogeneity with diffuse activity. Surveillance BMA was performed and revealed a significant decrease in the previously predominant lymphocyte population (15–20%). Flow cytometry findings were likewise compatible with the histopathology, revealing a mature B-cell neoplasm.

**Figure 3:**
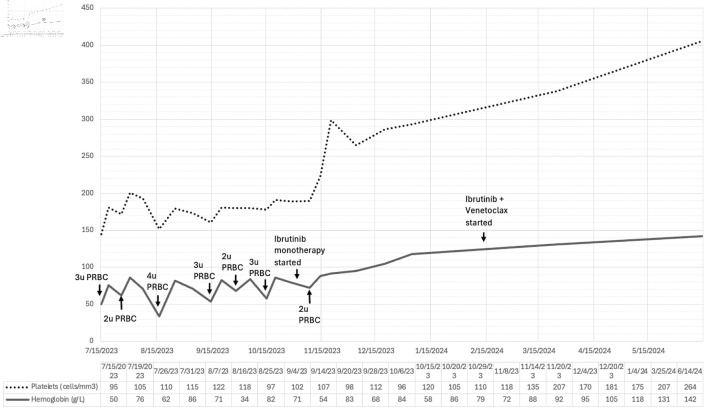
Temporal evolution of hemoglobin and platelet counts in relation to blood transfusion and initiation of treatment with ibrutinib monotherapy, followed by ibrutinib and venetoclax combination. PRBC: packed red blood cell.

Both ibrutinib and venetoclax were given for a total of 15 cycles of treatment (3-month ibrutinib lead-in followed by 12 months of ibrutinib and venetoclax). Her latest CBC showed a hemoglobin level of 142 g/L, hematocrit of 44%, WBC of 6,200 cells/mm^3^, with absolute lymphocyte count of 3,162 cells/mm^3^, and a platelet count of 264,000 cells/mm^3^. The temporal evolution of her hemoglobin and platelet counts prior to, and during treatment were shown in Figure 3. For now, she is being monitored regularly every 3 months, including her CBC, and serum chemistry.

## Discussion

CLL is a slowly progressive lymphoproliferative disorder that primarily affects older adults and shows a marked male predominance [1–3]. It is the most common form of leukemia among adults in Western countries, accounting for approximately 30% of cases. In contrast, CLL is considerably less frequent among individuals of Asian descent, with an incidence of only 2–3% [4, 6]. The disease occurs rarely in AYA, where it represents less than 1% of all CLL cases [2, 3, 5].

The diagnosis of SLL/CLL begins with a thorough history and physical examination. Patients may present with constitutional symptoms such as low-grade fever, weight loss, anorexia, lymphadenopathy, hepatosplenomegaly, and exertional dyspnea [1, 4]. SLL and CLL are considered different clinical manifestations of the same disease entity, distinguished primarily by the site of involvement and diagnostic criteria [1, 4]. SLL is characterized by predominant lymph node involvement with minimal peripheral blood lymphocytosis, whereas CLL is defined by persistent lymphocytosis exceeding 5,000/µL for at least 3 months, often accompanied by nodal, splenic, and bone marrow involvement [1, 5]. Treatment for CLL is initiated in the presence of bulky disease (splenomegaly > 6 cm or lymph nodes > 10 cm), progressive cytopenias, threatened end-organ function, refractory autoimmune cytopenias, or symptomatic disease, including fatigue, night sweats, or unintentional weight loss exceeding 10% within 6 months [1, 7].

Most clinical studies in CLL have focused on elderly populations, resulting in limited data on outcomes among AYA patients. Emerging evidence suggests that younger patients may harbor distinct genetic and molecular characteristics, including a higher frequency of adverse chromosomal abnormalities and gene mutations [2, 3, 8]. Cherng et al demonstrated that younger age, the presence of del(11q) or del(17p), complex karyotype, and CD38 positivity were associated with inferior outcomes, including shorter time to disease progression and increased risk of Richter transformation [8].

CLL cell proliferation primarily occurs within lymph node microanatomical structures known as pseudofollicles or proliferation centers [9, 10]. Disease survival and expansion are driven by interactions with the tumor microenvironment, including chemokines, toll-like receptor ligands, and accessory immune cells, with B-cell receptor (BCR) signaling serving as the central driver of nodal proliferation [9, 10]. Bruton’s tyrosine kinase (BTK) plays a critical role in this pathway by mediating downstream BCR signaling and facilitating CLL cell survival within the microenvironment [9]. Ibrutinib, the first-in-class irreversible BTK inhibitor, exerts its effect by covalently binding to the cysteine-481 residue within the BTK active site, thereby inhibiting kinase activity [9–11]. The introduction of ibrutinib has profoundly transformed the treatment landscape of CLL.

In the RESONATE-2 trial, patients with high-risk genetic features, including del(11q) or unmutated immunoglobulin heavy chain variable region (IGHV), demonstrated significantly superior long-term outcomes with single-agent ibrutinib compared with chlorambucil [8, 10]. Updated 7-year follow-up data from RESONATE-2 revealed an unprecedented overall survival rate of 78%, reinforcing the role of ibrutinib as frontline therapy for patients with high-risk disease [11]. However, concerns regarding long-term toxicity, financial burden, and cumulative treatment exposure—particularly in younger patients—have fueled growing interest in oral, fixed-duration treatment strategies.

At present, there are no locally established guidelines for the management of CLL/SLL in the Philippines. In this case, treatment decisions were guided by recommendations from the International Workshop on Chronic Lymphocytic Leukemia (iwCLL). Venetoclax is a selective inhibitor of the antiapoptotic protein BCL-2, which promotes apoptosis by displacing pro-apoptotic proteins and triggering downstream cell death pathways [12–14]. Venetoclax has demonstrated substantial efficacy in the treatment of CLL and SLL; however, its use is associated with hematologic toxicities such as neutropenia, thrombocytopenia, and anemia, which may increase the risk of infection and bleeding [13]. Tumor lysis syndrome (TLS) is a potentially life-threatening complication resulting from rapid leukemic cell breakdown, leading to metabolic derangements and renal dysfunction, and therefore necessitating careful risk stratification, prophylaxis, and close monitoring during therapy initiation [13].

Ibrutinib and venetoclax exert synergistic effects by targeting distinct disease compartments. Ibrutinib disrupts BCR signaling and mobilizes CLL cells from lymphoid tissues into the peripheral blood, thereby increasing their susceptibility to venetoclax-induced apoptosis [12, 14]. Evidence from the CAPTIVATE study demonstrated that first-line treatment with a fixed-duration regimen consisting of three cycles of ibrutinib lead-in followed by 12 cycles of combined ibrutinib and venetoclax achieved high complete remission rates of approximately 56% among high-risk patients, with undetectable minimal residual disease (uMRD) rates of 81% in peripheral blood and 41% in bone marrow [11, 12]. Updated results from CAPTIVATE further showed an 84% 4-year rate of freedom from next treatment, highlighting the regimen’s ability to induce deep, durable remissions and prolonged progression-free survival [12, 14].

Despite generally favorable tolerability, treatment with ibrutinib and venetoclax may be discontinued due to adverse events or financial constraints, particularly in resource-limited settings. Data on dose-reduction strategies in CLL remain limited. Chen et al evaluated the pharmacokinetics and pharmacodynamics of dose-reduced ibrutinib and demonstrated that following an initial cycle at the standard dose of 420 mg daily, subsequent cycles could be administered at lower doses without loss of biological activity [15]. Similarly, dose reduction of venetoclax has been proposed as a strategy to reduce treatment costs by 50% or more, thereby alleviating financial burden and potentially improving adherence. Reduced dosing may also mitigate toxicity while preserving clinical benefit. In a study by Marin et al, disease control—manifested by improvement in B symptoms and reduction in treatment-related adverse effects—was observed with daily venetoclax doses ranging from 100 to 200 mg [16]. These findings suggest that low-dose venetoclax may offer a balance between efficacy, tolerability, cost reduction, and quality-of-life preservation, although larger prospective studies are required to confirm long-term safety and effectiveness.

Data on CLL in AYAs remain sparse, but available evidence suggests a distinct disease profile. Published case series and registry analyses consistently indicate that AYA patients present with more advanced Rai or Binet stage disease, bulky lymphadenopathy, and a higher prevalence of adverse biological features—including unmutated IGHV, ZAP-70 positivity, and high-risk cytogenetic abnormalities—despite generally preserved performance status [8, 16]. Although overall survival in AYA patients is often favorable compared with older cohorts, progression-free survival tends to be shorter, reflecting earlier need for treatment and a more aggressive disease course. Importantly, contemporary reports suggest that CLL biology in AYA patients more closely resembles high-risk adult CLL rather than an indolent variant, raising concerns regarding cumulative treatment toxicity, fertility preservation, and the risk of secondary malignancies over decades of survivorship. Current management recommendations for this population continue to follow adult iwCLL guidelines; however, the literature repeatedly underscores the absence of AYA-specific prospective trials and highlights the need for individualized treatment strategies that incorporate targeted therapies, survivorship planning, and psychosocial considerations unique to younger patients [8].

### Conclusions

CLL mainly affects the elderly and is exceptionally rare in young adults, posing unique clinical challenges. While newer therapies and supportive care have improved outcomes, further studies are needed to optimize treatment for this population, particularly in Asia, with focus on dose adjustments in resource-limited settings to balance safety, efficacy, and quality of life.

This case underscores the importance of early diagnosis and thoughtful treatment selection in high-risk young patients. A fixed-duration, reduced-dose ibrutinib–venetoclax regimen shows promise as a safe and effective option for AYA CLL.

### Learning points

CLL primarily affects the elderly and is extremely rare in young adults, thus, requiring thorough investigation, and presenting distinct challenges.

Significant considerations include appropriate dosage adjustments in a resource-limited setting while gearing towards balancing safety and efficacy, maximizing outcomes, and enhancing quality of life.

Currently, there are no studies supporting reduced-dosage and fixed-duration regimen for CLL.

Fixed-duration, reduced-dosage ibrutinib and venetoclax regimen appears promising as a safe and effective treatment option for CLL patients in the AYA group, especially in a resource-constrained setting.

## Data Availability

All data supporting the findings of this case report are included within the article. Additional anonymized information may be made available from the corresponding author upon reasonable request, subject to institutional and ethical review.
